# Supplementary material for: Pyruvate Dehydrogenase Kinase 1 inhibition mediated oxidative phosphorylation enhancement in cartilage promotes osteoarthritis progression
Source: BMC Musculoskelet Disord. 2023 Jul 20;24:597. doi: 10.1186/s12891-023-06585-6 (PMC10357736; doi:10.1186/s12891-023-06585-6)
Supplement: Supplementary file 1 — Additional file 1. [file 12891_2023_6585_MOESM1_ESM.zip › MF.Gsea.1653627316242/GOMF_AMINO_ACID_SODIUM_SYMPORTER_ACTIVITY.html]

Details for gene set GOMF\_AMINO\_ACID\_SODIUM\_SYMPORTER\_ACTIVITY[GSEA]

|  || Dataset | 55457\_collapsed\_to\_symbols.cls.cls #OA\_versus\_normal.cls.cls #OA\_versus\_normal\_repos |
| Phenotype | cls.cls#OA\_versus\_normal\_repos |
| Upregulated in class | normal |
| GeneSet | GOMF\_AMINO\_ACID\_SODIUM\_SYMPORTER\_ACTIVITY |
| Enrichment Score (ES) | -0.51294374 |
| Normalized Enrichment Score (NES) | -1.4954324 |
| Nominal p-value | 0.04020979 |
| FDR q-value | 0.18227041 |
| FWER p-Value | 1.0 |
Table: GSEA Results Summary

  

Fig 1: Enrichment plot: GOMF\_AMINO\_ACID\_SODIUM\_SYMPORTER\_ACTIVITY      
 Profile of the Running ES Score & Positions of GeneSet Members on the Rank Ordered List

  

| SYMBOL | TITLE | RANK IN GENE LIST | RANK METRIC SCORE | RUNNING ES | CORE ENRICHMENT || 1 | SLC6A12 | solute carrier family 6 member 12 [Source:HGNC Symbol;Acc:HGNC:11045] | 4093 | 0.147 | -0.2849 | No |
| 2 | SLC6A1 | solute carrier family 6 member 1 [Source:HGNC Symbol;Acc:HGNC:11042] | 6970 | -0.078 | -0.4907 | Yes |
| 3 | SLC1A2 | solute carrier family 1 member 2 [Source:HGNC Symbol;Acc:HGNC:10940] | 7026 | -0.082 | -0.4767 | Yes |
| 4 | SLC6A5 | solute carrier family 6 member 5 [Source:HGNC Symbol;Acc:HGNC:11051] | 7439 | -0.114 | -0.4832 | Yes |
| 5 | SLC6A13 | solute carrier family 6 member 13 [Source:HGNC Symbol;Acc:HGNC:11046] | 7463 | -0.115 | -0.4592 | Yes |
| 6 | SLC1A1 | solute carrier family 1 member 1 [Source:HGNC Symbol;Acc:HGNC:10939] | 8157 | -0.167 | -0.4755 | Yes |
| 7 | SLC1A7 | solute carrier family 1 member 7 [Source:HGNC Symbol;Acc:HGNC:10945] | 8504 | -0.194 | -0.4589 | Yes |
| 8 | SLC6A9 | solute carrier family 6 member 9 [Source:HGNC Symbol;Acc:HGNC:11056] | 8704 | -0.209 | -0.4275 | Yes |
| 9 | SLC1A3 | solute carrier family 1 member 3 [Source:HGNC Symbol;Acc:HGNC:10941] | 9656 | -0.288 | -0.4369 | Yes |
| 10 | SLC6A7 | solute carrier family 6 member 7 [Source:HGNC Symbol;Acc:HGNC:11054] | 10185 | -0.339 | -0.4020 | Yes |
| 11 | SLC38A1 | solute carrier family 38 member 1 [Source:HGNC Symbol;Acc:HGNC:13447] | 10617 | -0.385 | -0.3493 | Yes |
| 12 | SLC1A6 | solute carrier family 1 member 6 [Source:HGNC Symbol;Acc:HGNC:10944] | 10626 | -0.386 | -0.2636 | Yes |
| 13 | SLC6A15 | solute carrier family 6 member 15 [Source:HGNC Symbol;Acc:HGNC:13621] | 10966 | -0.426 | -0.1946 | Yes |
| 14 | SLC6A20 | solute carrier family 6 member 20 [Source:HGNC Symbol;Acc:HGNC:30927] | 10982 | -0.428 | -0.1000 | Yes |
| 15 | SLC38A2 | solute carrier family 38 member 2 [Source:HGNC Symbol;Acc:HGNC:13448] | 11722 | -0.536 | -0.0373 | Yes |
| 16 | SLC6A11 | solute carrier family 6 member 11 [Source:HGNC Symbol;Acc:HGNC:11044] | 11916 | -0.575 | 0.0764 | Yes |
Table: GSEA details [plain text format]

  

Fig 2: GOMF\_AMINO\_ACID\_SODIUM\_SYMPORTER\_ACTIVITY      
 Blue-Pink O' Gram in the Space of the Analyzed GeneSet

  

Fig 3: GOMF\_AMINO\_ACID\_SODIUM\_SYMPORTER\_ACTIVITY: Random ES distribution      
 Gene set null distribution of ES for **GOMF\_AMINO\_ACID\_SODIUM\_SYMPORTER\_ACTIVITY**
